# Development and validation of a risk nomogram for failed conversion from labor epidural analgesia to cesarean epidural anesthesia

**DOI:** 10.3389/fmed.2026.1796836

**Published:** 2026-06-18

**Authors:** Guangyuan Su, Xue Cheng, Yanheng Jin, Yibing Jing, Lizhong Yu, Hong Sun, Chao Han

**Affiliations:** 1 Department of Anesthesiology, Affiliated Yixing Hospital of Jiangsu University, Yixing, China; 2 Clinical Medical College, Nanjing Medical University, Nanjing, China; 3 Department of Anesthesiology, Yixing Hospital of Traditional Chinese Medicine, Yixing, China

**Keywords:** cesarean epidural anesthesia, conversion failure, labor epidural analgesia, nomogram, predictive model

## Abstract

**Purpose:**

This study aimed to develop and validate a risk prediction nomogram for failed conversion from epidural labor analgesia to cesarean epidural anesthesia.

**Patients and methods:**

Parturients undergoing non-immediate cesarean section after epidural labor analgesia between January 2022 and December 2024 across two centers were retrospectively analyzed (model-development cohort: 389; external validation cohort: 129). Potential risk factors were screened by univariate analysis in the training cohort, and six independent predictors were identified using multivariable logistic regression. A predictive model was then developed based on these predictors and validated using external data. Model performance was evaluated by discrimination (AUROC), calibration, and internal/external validation.

**Results:**

The conversion failure rate was 18.5% in the model-development cohort and 18.6% in the external validation cohort. Six predictors were incorporated into the nomogram: obstetric anesthesiologist involvement, 10-min post-analgesia VAS score, breakthrough pain, frequent rescue epidural top-ups, asymmetric sensory blockade, and programmed intermittent epidural bolus (PIEB) pump mode. The model showed good discrimination in both the training (AUROC 0.89, 95% CI 0.85–0.94) and external validation (AUROC 0.89, 95% CI 0.83–0.96) cohorts, with acceptable calibration.

**Conclusion:**

We developed and externally validated a clinically interpretable nomogram for predicting failed labor-to-cesarean epidural conversion. Incorporating six routinely available variables, this tool may help convert early clinical signs of inadequate epidural function into a quantitative decision aid for anesthetic planning.

## 1 Introduction

Labor pain is among the most intense pain experiences women may encounter during their lifetime, ranking second only to burning pain on the medical pain intensity scale. The physiological response to labor pain—including marked stimulation of respiratory and circulatory systems, activation of hypothalamic autonomic centers and neuroendocrine pathways, and engagement of limbic structures—can trigger adverse consequences for both maternal and fetal/neonatal health. Additionally, the psychodynamic responses, such as anxiety and apprehension, may further exacerbate these effects (1). Consequently, effective pain relief is a cornerstone of modern obstetric care during childbirth.

Labor neuraxial analgesia (LNA) is currently accepted as the gold standard for labor pain management, with techniques including epidural analgesia (EA), combined spinal-epidural (CSE), and dural puncture epidural (DPE) (2–4). The World Health Organization (WHO) recommends LNA for healthy pregnant women as the most effective method of intrapartum pain relief (4). A Cochrane systematic review confirmed that LNA provides superior pain relief compared to non-epidural methods without increasing the risk of cesarean section (5).

When an emergency cesarean section becomes necessary for a parturient receiving epidural labor analgesia, extending anesthesia through the indwelling epidural catheter is the preferred approach. This strategy preserves the key advantage of neuraxial analgesia—avoiding the potentially catastrophic risks of general anesthesia, including difficult airway management, reduced maternal oxygen reserve, and pulmonary aspiration (6).

However, this conversion is not universally successful. Reported failure rates range from 0% to 21%, with considerable heterogeneity across studies due to differences in failure definitions, analgesic techniques, and institutional protocols (7, 8). Failed conversion has significant clinical consequences: it causes maternal anxiety and discomfort, delays surgical initiation, and may compromise fetal safety in time-sensitive situations. For anesthesiologists, failed conversion necessitates rescue strategies such as spinal anesthesia or conversion to general anesthesia, both of which carry additional risks in the context of partial epidural blockade (9). Therefore, preemptive identification of patients at high risk for conversion failure is clinically imperative.

While previous studies have identified multiple risk factors for conversion failure (8, 10–15), translating these factors into real-time clinical decisions remains challenging. Nomograms integrate multiple predictor variables to quantify individualized risk probabilities (16), offering a practical tool for bedside decision-making.

## 2 Materials and methods

### 2.1 Study design and patients

The study was conducted in two phases: model derivation using retrospective data from parturients who underwent conversion from epidural labor analgesia to CS epidural anesthesia at Yixing People’s Hospital (January 2022–December 2023), followed by external validation with a retrospectively collected cohort from Yixing Hospital of Traditional Chinese Medicine (July 2023–December 2024).

Inclusion criteria were: (1) parturients who received epidural labor analgesia; (2) subsequently underwent non-immediate (Category 2) cesarean section; (3) age ≥ 18 years; (4) gestational age ≥ 37 weeks. Category 1 cesarean sections (immediate threat to life) were excluded as they routinely received general anesthesia per institutional protocol.

Exclusion criteria were as follows: (1) serious organic pathologies involving the heart, lungs, or brain; (2) direct referral for general anesthesia by obstetric teams; (3) blood or cerebrospinal fluid (CSF) aspiration through the epidural catheter; (4) epidural catheter malposition; (5) abnormal pelvic measurement; and (6) incomplete clinical information.

### 2.2 Ethics approval

This study was approved by the Ethics Committee of Yixing People’s Hospital (approval number: IRB-2022-S-092; approval date: 15 March 2022; chairperson: Dr. Fan Yongyan). As Yixing Hospital of Traditional Chinese Medicine served as a validation center with fully anonymized data and no additional interventions, the primary ethics committee approval covered both institutions following a data transfer agreement. The requirement for informed consent was waived due to the retrospective study design and use of previously collected clinical data. All patient information was anonymized and kept confidential prior to analysis. Due to the retrospective nature of the study, prospective registration was not required by the institutional ethics committee.

### 2.3 Data collection

Based on a review of original observational studies, 38 potential risk factors were identified for investigation. These explicitly referenced variables included maternal characteristics and preoperative comorbidities (maternal age, body mass index prior to conception and at delivery, gestational weight gain, number of pregnancies, gravidity, parity, gestational age, fundal height, abdominal circumference, cervical dilation at admission, pregnancy-induced hypertension, gestational diabetes mellitus, history of uterine surgery) (12, 13); history of operation and neuraxial anesthesia (8, 12); fetal characteristics and uterine contraction initiation (7, 8); epidural puncture details (patient position, vertebral level of insertion, puncture approach, puncture technology, direction and length of catheter, symptoms of neurological irritation, number of attempts) (17); pain management parameters [type of labor analgesia, drug delivery method (PIEB vs. PCEA), VAS score before and 10 min after labor analgesia, number of rescue epidural top-ups, breakthrough pain, asymmetric sensory blockade, total time and dose of local anesthetic] (12, 13, 18); labor-related events (Bromage score, intrapartum fever, hypotension) (12); anesthesiologist type (19); and indication for cesarean delivery. All information was obtained from the hospitals’ electronic database.

### 2.4 Anesthesia protocol

Labor analgesia modality—epidural analgesia, combined spinal-epidural (CSE), or dural puncture epidural (DPE)—was at the discretion of the attending anesthesiologist. All parturients received ropivacaine 1 mg/mL with sufentanil 0.25 μg/mL, with an induction dose of 5 mL, patient-controlled bolus 5 mL, and lockout interval 30 min. Maintenance delivery was either patient-controlled epidural analgesia (PCEA), with a continuous background infusion at 10 mL/h, or programmed intermittent epidural bolus (PIEB), delivering a bolus of 10 mL every 60 min with no background infusion; the choice was at the discretion of the attending anesthesiologist.

At the decision for cesarean section, epidural extension was achieved with 2% lidocaine 10 mL administered in divided doses: 3 mL test dose followed by 7 mL after 5 min if no signs of intrathecal injection. This 10 mL represents the test and loading dose only; additional doses of 2% lidocaine (5 mL increments) were administered as needed based on sequential block height assessment to achieve adequate surgical anesthesia. Crucially, following this initial 10 mL test and loading dose, if the sensory block level remained inadequate for surgery, additional local anesthetics were sequentially titrated in 3–5 mL increments based on block progression. Conversion failure was strictly defined only when maximal optimal epidural titrations failed to provide adequate surgical conditions.

### 2.5 Definition of failed conversion

Conversion failure was defined as inadequate surgical anesthesia after epidural top-up requiring any of the following: (1) systemic adjuvant analgesics, (2) re-puncture for spinal anesthesia, or (3) conversion to general anesthesia. Category 1 cesarean sections were excluded as they routinely received general anesthesia per institutional protocol. This definition was consistently applied throughout the study.

### 2.6 Definition of anesthesiologist type

Obstetric anesthesiologist was defined as attending anesthesiologists dedicated to the obstetric unit during weekday daytime shifts (8:00–17:00, Monday to Friday). Non-obstetric anesthesiologists were those covering the obstetric unit during nights, weekends, or holidays as part of general on-call duties. This classification reflects institutional staffing patterns rather than individual competence.

### 2.7 Statistical analysis

R software (R Foundation for Statistical Computing, Vienna, Austria) was utilized for all analyses. Independent variables were analyzed according to predefined clinical categories. The model-development cohort comprised parturients who received epidural analgesia for labor and met inclusion/exclusion criteria at Yixing People’s Hospital between January 2022 and December 2023. The external validation cohort included retrospectively enrolled parturients from Yixing Hospital of Traditional Chinese Medicine between July 2023 and December 2024. Baseline characteristics of all 38 candidate variables in the model-development and external validation cohorts are provided in the Supplementary material (Supplementary Table 1).

Univariate analysis was initially performed to identify potential risk variables. Variables with *P* < 0.10 in univariate analysis were evaluated for multivariable modeling, and a clinically parsimonious six-predictor logistic regression model was retained for the nomogram. The 10-min post-analgesia VAS score was modeled as categorical dummy variables using ≤3 as the reference category, with separate estimates for 4–7 and ≥8. Multicollinearity was assessed using variance inflation factor (VIF), with VIF > 5 indicating collinearity. Complete-case analysis was performed; no patient record included in the final analysis had missing predictor or outcome data. Internal validation was performed using bootstrapping with 1,000 resamples to obtain optimism-corrected AUROC.

Model calibration was assessed using calibration plots, and clinical utility was evaluated through decision curve analysis (DCA). Statistical significance was defined as *P* < 0.05.

## 3 Results

### 3.1 Baseline characteristics

**FIGURE 1:**
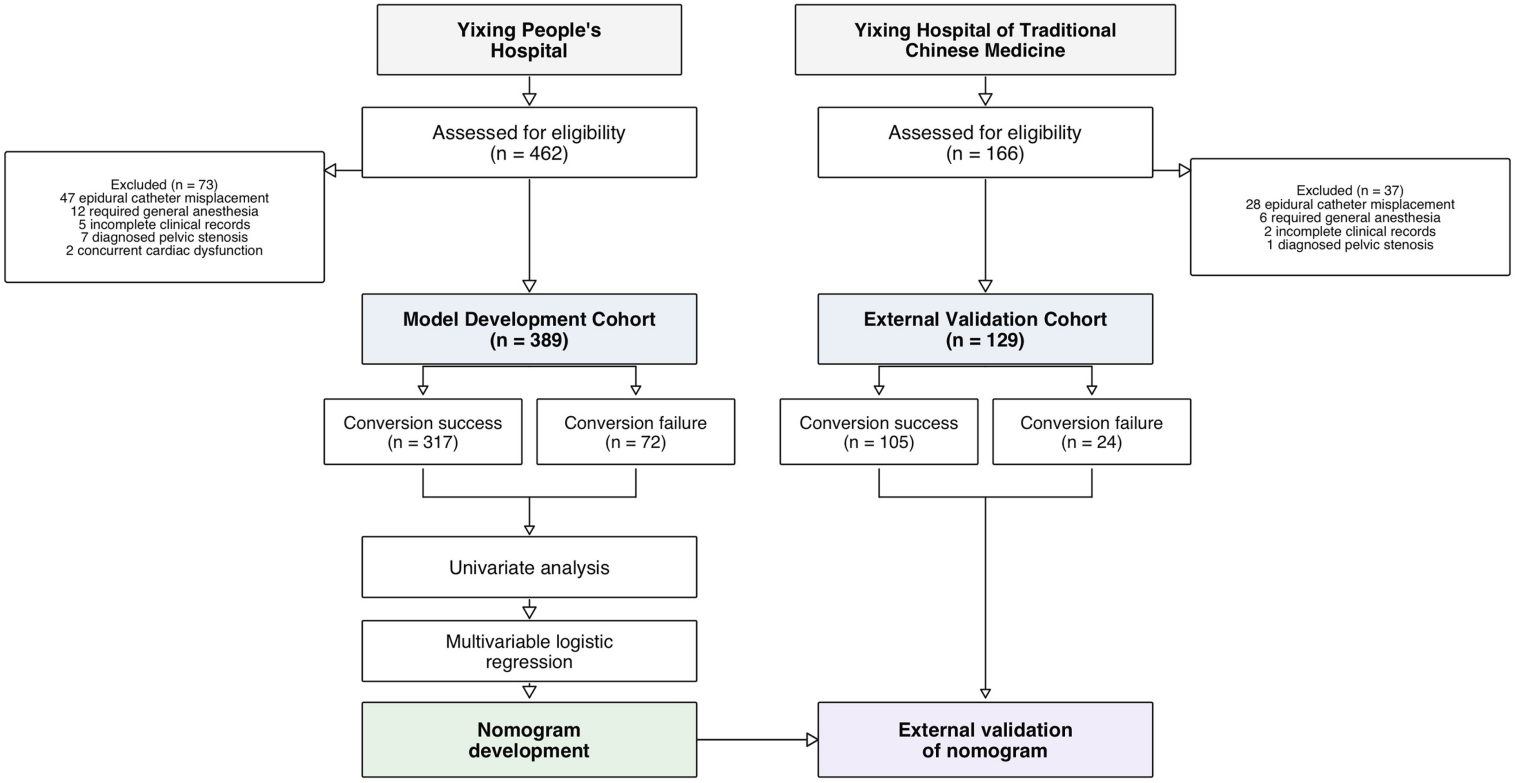
Flowchart of study population selection and model development/validation.

Among 462 parturients screened at Yixing People’s Hospital, 73 were excluded: 47 due to epidural catheter misplacement/dislodgement, 12 at obstetricians’ discretion for immediate general anesthesia, five owing to incomplete clinical records, seven with diagnosed pelvic stenosis, and two with concurrent cardiac dysfunction. The final model-development cohort comprised 389 parturients. Following the same inclusion and exclusion criteria, 129 parturients were enrolled from Yixing Hospital of Traditional Chinese Medicine as the external validation cohort. The research process is illustrated in Figure 1.

Among the 389 parturients in the model-development cohort, 317 (81.5%) had successful conversion and 72 (18.5%) experienced conversion failure. Reasons for failure included: 46 received adjuvant analgesics, 17 underwent re-puncture for spinal anesthesia, and nine were converted to general anesthesia. Baseline characteristics of the model-development cohort stratified by conversion outcome are presented in Table 1.

**TABLE 1:** Baseline characteristics of the model-development cohort stratified by conversion outcome.

Variable	Category	Total (*n* = 389)	Success (*n* = 317)	Failure (*n* = 72)	*P*-value
Maternal characteristics					
Pre-pregnancy BMI	<18.5	34 (8.7%)	29 (9.1%)	5 (6.9%)	0.943
	18.5–24.9	263 (67.6%)	212 (66.9%)	51 (70.8%)	–
	25.0–29.9	85 (21.9%)	70 (22.1%)	15 (20.8%)	–
	≥30	7 (1.8%)	6 (1.9%)	1 (1.4%)	–
BMI at end of pregnancy	<18.5	0 (0.0%)	0 (0.0%)	0 (0.0%)	0.599
	18.5–24.9	36 (9.3%)	29 (9.1%)	7 (9.7%)	–
	25.0–29.9	311 (79.9%)	256 (80.8%)	55 (76.4%)	–
	≥30	42 (10.8%)	32 (10.1%)	10 (13.9%)	–
Age	≤35 years	332 (85.3%)	267 (84.2%)	65 (90.3%)	0.190
	>35 years	57 (14.7%)	50 (15.8%)	7 (9.7%)	–
Gravidity	Primipara	329 (84.6%)	263 (83.0%)	66 (91.7%)	0.065
	Multipara	60 (15.4%)	54 (17.0%)	6 (8.3%)	–
Pregnancy-induced hypertension	No	352 (90.5%)	287 (90.5%)	65 (90.3%)	0.946
	Yes	37 (9.5%)	30 (9.5%)	7 (9.7%)	–
Gestational diabetes mellitus	No	325 (83.5%)	266 (83.9%)	59 (81.9%)	0.684
	Yes	64 (16.5%)	51 (16.1%)	13 (18.1%)	–
Previous abdominal surgery	No	366 (94.1%)	299 (94.3%)	67 (93.1%)	0.781
	Yes	23 (5.9%)	18 (5.7%)	5 (6.9%)	–
Previous epidural analgesia	No	378 (97.2%)	309 (97.5%)	69 (95.8%)	0.434
	Yes	11 (2.8%)	8 (2.5%)	3 (4.2%)	–
Fetal and obstetric factors					
Biparietal diameter	Standard	350 (90.0%)	290 (91.5%)	60 (83.3%)	0.102
	<Standard	5 (1.3%)	4 (1.3%)	1 (1.4%)	–
	>Standard	34 (8.7%)	23 (7.3%)	11 (15.3%)	–
Gestational weight gain	Moderate	291 (74.8%)	240 (75.7%)	51 (70.8%)	0.457
	Insufficient	26 (6.7%)	22 (6.9%)	4 (5.6%)	–
	Excessive	72 (18.5%)	55 (17.4%)	17 (23.6%)	–
Abdominal circumference	Standard	349 (89.7%)	288 (90.9%)	61 (84.7%)	0.224
	<Standard	9 (2.3%)	7 (2.2%)	2 (2.8%)	–
	>Standard	31 (8.0%)	22 (6.9%)	9 (12.5%)	–
Fundal height	32–38 cm	348 (89.5%)	286 (90.2%)	62 (86.1%)	0.365
	<32 cm	6 (1.5%)	4 (1.3%)	2 (2.8%)	–
	>38 cm	35 (9.0%)	27 (8.5%)	8 (11.1%)	–
Macrosomia	No	365 (93.8%)	298 (94.0%)	67 (93.1%)	0.786
	Yes	24 (6.2%)	19 (6.0%)	5 (6.9%)	–
Uterine contraction initiation	Spontaneous	247 (63.5%)	215 (67.8%)	32 (44.4%)	<0.001
	Drug induction	100 (25.7%)	71 (22.4%)	29 (40.3%)	–
	Artificial rupture of membranes	42 (10.8%)	31 (9.8%)	11 (15.3%)	–
Gestational age	37–40 weeks	307 (78.9%)	253 (79.8%)	54 (75.0%)	0.192
	40–42 weeks	72 (18.5%)	58 (18.3%)	14 (19.4%)	–
	>42 weeks	10 (2.6%)	6 (1.9%)	4 (5.6%)	–
Cervical dilation	>2 cm	300 (77.1%)	248 (78.2%)	52 (72.2%)	0.273
	0–2 cm	89 (22.9%)	69 (21.8%)	20 (27.8%)	–
Indication for cesarean delivery	Fetal distress	147 (37.8%)	120 (37.9%)	27 (37.5%)	0.849
	Abnormal fetal position	175 (45.0%)	144 (45.4%)	31 (43.1%)	–
	Retention	67 (17.2%)	53 (16.7%)	14 (19.4%)	–
Epidural puncture and catheter factors					
Epidural puncture space	L2-3	299 (76.9%)	252 (79.5%)	47 (65.3%)	0.010
	L3-4	90 (23.1%)	65 (20.5%)	25 (34.7%)	–
Obstetric anesthesiologist	No	169 (43.4%)	123 (38.8%)	46 (63.9%)	<0.001
	Yes	220 (56.6%)	194 (61.2%)	26 (36.1%)	–
Epidural puncture approach	Median approach	198 (50.9%)	163 (51.4%)	35 (48.6%)	0.667
	Lateral approach	191 (49.1%)	154 (48.6%)	37 (51.4%)	–
Position during puncture	Sitting	2 (0.5%)	2 (0.6%)	0 (0.0%)	0.931
	Left lateral decubitus	218 (56.0%)	178 (56.2%)	40 (55.6%)	–
	Right lateral decubitus	169 (43.4%)	137 (43.2%)	32 (44.4%)	–
Direction of epidural catheter	Cranial	338 (86.9%)	274 (86.4%)	64 (88.9%)	0.578
	Caudal	51 (13.1%)	43 (13.6%)	8 (11.1%)	–
Catheter insertion length	≥3 cm	372 (95.6%)	306 (96.5%)	66 (91.7%)	0.102
	<3 cm	17 (4.4%)	11 (3.5%)	6 (8.3%)	–
Puncture technology	Resistance disappearance	354 (91.0%)	289 (91.2%)	65 (90.3%)	0.812
	Negative pressure	35 (9.0%)	28 (8.8%)	7 (9.7%)	–
Symptoms of neurological irritation	No	371 (95.4%)	305 (96.2%)	66 (91.7%)	0.117
	Yes	18 (4.6%)	12 (3.8%)	6 (8.3%)	–
Attempts to insert the epidural catheter	1–2	373 (95.9%)	306 (96.5%)	67 (93.1%)	0.190
	≥3	16 (4.1%)	11 (3.5%)	5 (6.9%)	–
Labor analgesia and intrapartum factors					
Labor analgesia mode	Epidural	213 (54.8%)	172 (54.3%)	41 (56.9%)	0.782
	Combined spinal-epidural	148 (38.0%)	123 (38.8%)	25 (34.7%)	–
	Dural puncture epidural	28 (7.2%)	22 (6.9%)	6 (8.3%)	–
VAS before labor analgesia	≤3	83 (21.3%)	68 (21.5%)	15 (20.8%)	0.951
	4–7	118 (30.3%)	97 (30.6%)	21 (29.2%)	–
	≥8	188 (48.3%)	152 (47.9%)	36 (50.0%)	–
VAS at 10 min post-analgesia	≤3	322 (82.8%)	287 (90.5%)	35 (48.6%)	<0.001
	4–7	63 (16.2%)	28 (8.8%)	35 (48.6%)	–
	≥8	4 (1.0%)	2 (0.6%)	2 (2.8%)	–
Breakthrough pain	No	332 (85.3%)	288 (90.9%)	44 (61.1%)	<0.001
	Yes	57 (14.7%)	29 (9.1%)	28 (38.9%)	–
Rescue epidural top-ups	<2	345 (88.7%)	292 (92.1%)	53 (73.6%)	<0.001
	≥ 2	44 (11.3%)	25 (7.9%)	19 (26.4%)	–
Pump mode	PCEA	157 (40.4%)	113 (35.6%)	44 (61.1%)	<0.001
	PIEB	232 (59.6%)	204 (64.4%)	28 (38.9%)	–
Time from epidural analgesia to cesarean section	<3 h	77 (19.8%)	66 (20.8%)	11 (15.3%)	0.515
	3–6 h	297 (76.3%)	238 (75.1%)	59 (81.9%)	–
	>6 h	15 (3.9%)	13 (4.1%)	2 (2.8%)	–
Bromage score 30 min after analgesia	0	371 (95.4%)	300 (94.6%)	71 (98.6%)	0.216
	≥1	18 (4.6%)	17 (5.4%)	1 (1.4%)	–
Fever	No	336 (86.4%)	275 (86.8%)	61 (84.7%)	0.651
	Yes	53 (13.6%)	42 (13.2%)	11 (15.3%)	–
Hypotension	No	333 (85.6%)	266 (83.9%)	67 (93.1%)	0.046
	Yes	56 (14.4%)	51 (16.1%)	5 (6.9%)	–
Total dose of local anesthetic during labor	≤30 ml	63 (16.2%)	51 (16.1%)	12 (16.7%)	0.817
	30–70 ml	191 (49.1%)	158 (49.8%)	33 (45.8%)	–
	≥70 ml	135 (34.7%)	108 (34.1%)	27 (37.5%)	–
Asymmetric sensory blockade	No	371 (95.4%)	310 (97.8%)	61 (84.7%)	<0.001
	Yes	18 (4.6%)	7 (2.2%)	11 (15.3%)	–

Values are presented as *n* (%). *P*-values compare the success and failure groups using Pearson chi-square or Fisher exact tests as appropriate.

### 3.2 Development of the prediction model

In univariate logistic regression analysis of 38 potential risk factors, variables with *P* < 0.10 were evaluated for multivariable modeling (Table 2). The final six-predictor model showed no significant multicollinearity, with a maximum adjusted GVIF of 1.12.

**TABLE 2:** Univariate and multivariate logistic regression analysis.

Variable/comparison	Univariate OR (95% CI)	Univariate *P*	Multivariate OR (95% CI)	Multivariate *P*	Final model
Gravidity: multipara vs. primipara	0.44 (0.18–1.07)	0.071	–	–	–
Uterine contraction initiation: drug induction vs. spontaneous	2.74 (1.55–4.85)	<0.001	–	–	–
Artificial rupture of membranes vs. spontaneous	2.38 (1.09–5.21)	0.029	–	–	–
Epidural puncture space: L3-4 vs. L2-3	2.06 (1.18–3.60)	0.011	–	–	–
Obstetric anesthesiologist: yes vs. no	0.36 (0.21–0.61)	<0.001	0.38 (0.19–0.77)	0.007	Yes
Catheter insertion length: <3 vs. ≥3 cm	2.53 (0.90–7.08)	0.077	–	–	–
VAS at 10 min post-analgesia: 4–7 vs. ≤3	10.25 (5.58–18.83)	<0.001	16.34 (7.29–36.61)	<0.001	Yes
≥8 vs. ≤3	8.20 (1.12–60.06)	0.038	20.24 (2.27–180.69)	0.007	Yes
Breakthrough pain: yes vs. no	6.32 (3.44–11.61)	<0.001	10.94 (4.76–25.12)	<0.001	Yes
Rescue epidural top-ups: ≥2 vs. <2	4.19 (2.15–8.14)	<0.001	6.93 (2.71–17.73)	<0.001	Yes
Pump mode: PIEB vs. PCEA	0.35 (0.21–0.60)	<0.001	0.38 (0.19–0.75)	0.005	Yes
Hypotension: yes vs. no	0.39 (0.15–1.01)	0.053	–	–	–
Asymmetric sensory blockade: yes vs. no	7.99 (2.98–21.42)	<0.001	8.71 (2.33–32.52)	0.001	Yes

From the initial 38 candidate variables identified through literature review, variables with *P* < 0.10 in univariate analysis were evaluated for multivariable modeling. The final six-predictor model is shown in the multivariate columns. The 10-min post-analgesia VAS score was entered as categorical dummy variables with ≤3 as the reference category. Maximum adjusted GVIF was 1.12, indicating no significant multicollinearity.

The final multivariable logistic regression model retained six predictors for failed conversion (Table 2): obstetric anesthesiologist involvement (OR 0.38, 95% CI 0.19–0.77, *P* = 0.007), VAS at 10 min post-analgesia (4–7 vs. ≤3: OR 16.34, 95% CI 7.29–36.61, *P* < 0.001; ≥8 vs. ≤3: OR 20.24, 95% CI 2.27–180.69, *P* = 0.007), breakthrough pain (OR 10.94, 95% CI 4.76–25.12, *P* < 0.001), rescue epidural top-ups ≥ 2 (OR 6.93, 95% CI 2.71–17.73, *P* < 0.001), asymmetric sensory blockade (OR 8.71, 95% CI 2.33–32.52, *P* = 0.001), and pump mode (PIEB vs. PCEA: OR 0.38, 95% CI 0.19–0.75, *P* = 0.005). The model showed good fit (Nagelkerke R^2^ = 0.51, Hosmer-Lemeshow *P* = 0.890, Brier score = 0.088, maximum adjusted GVIF = 1.12, EPV per model degree of freedom = 10.3).

**FIGURE 2:**
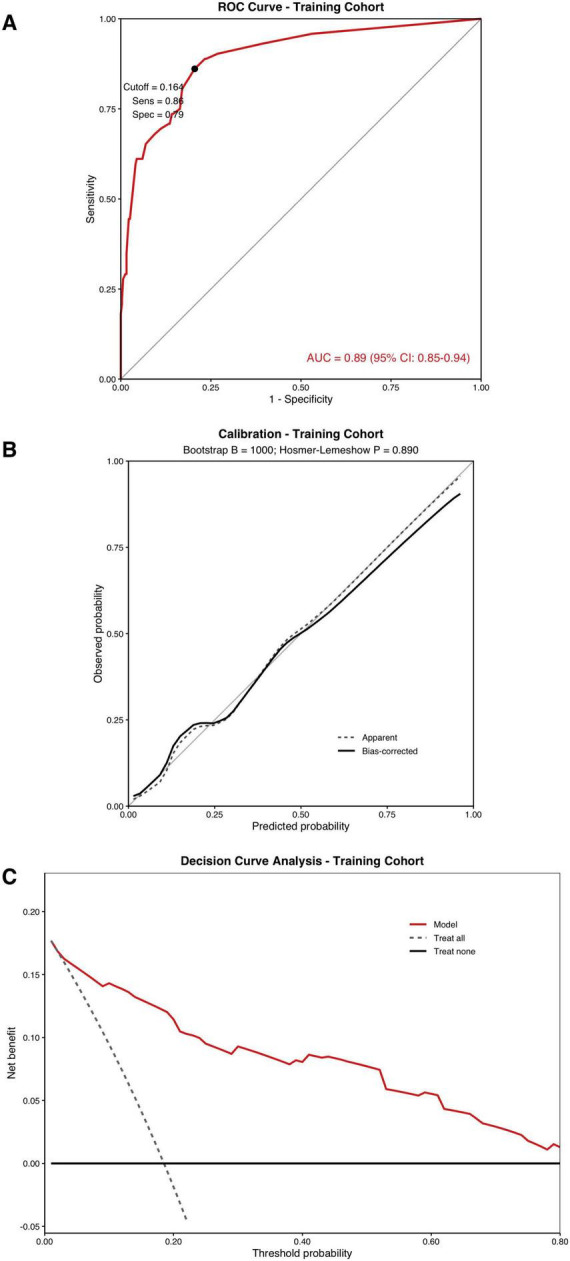
Performance of the prediction model in the training cohort. **(A)** Receiver operating characteristic (ROC) curve with AUROC of 0.89 (95% CI 0.85–0.94). **(B)** Calibration plot showing agreement between predicted and observed probabilities (Bootstrap B = 1,000; Hosmer-Lemeshow *P* = 0.890). **(C)** Decision curve analysis demonstrating net benefit across clinically relevant threshold probabilities.

The model demonstrated good discriminative performance, with an AUROC of 0.89 (95% CI: 0.85–0.94) in the model-development cohort (Figure 2A). Calibration analysis via the Hosmer-Lemeshow test was non-significant (*P* = 0.890), and the calibration curve showed close agreement between predicted and observed probabilities (Figure 2B). Decision curve analysis (DCA) demonstrated net benefit across clinically relevant risk thresholds (Figure 2C).

### 3.3 Validation of the prediction model

**FIGURE 3:**
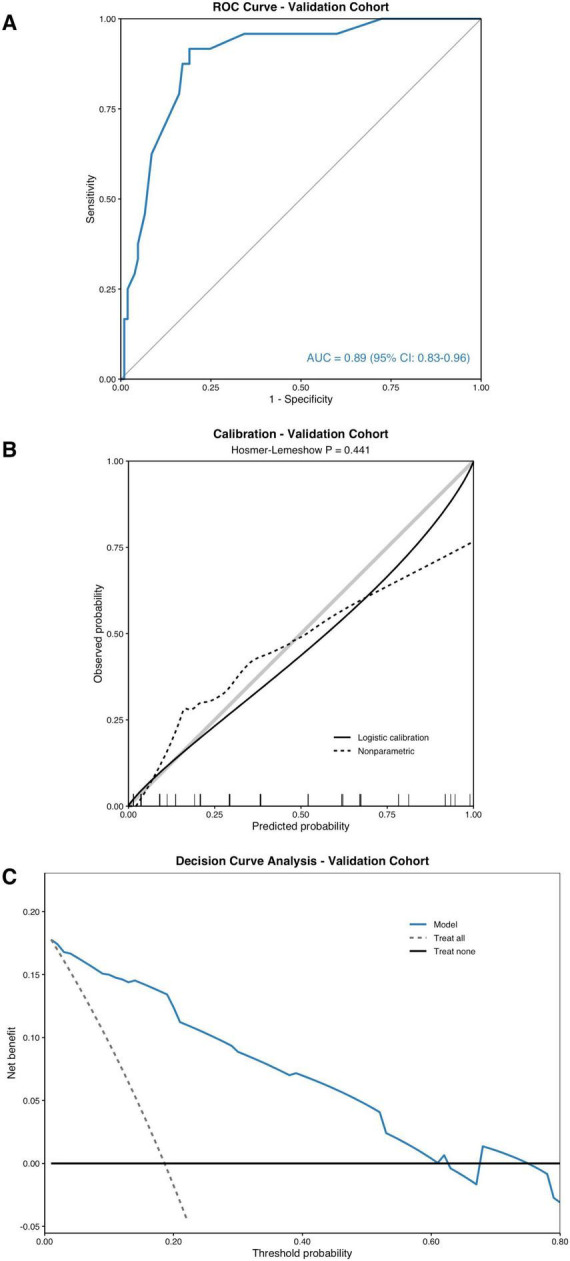
Performance of the prediction model in the validation cohort. **(A)** ROC curve with AUROC of 0.89 (95% CI 0.83–0.96). **(B)** Calibration plot (Hosmer-Lemeshow *P* = 0.441). **(C)** Decision curve analysis.

In the external validation cohort, the model maintained good discriminative performance with an AUROC of 0.89 (95% CI: 0.83–0.96) (Figure 3A). Calibration analysis via the Hosmer-Lemeshow test was non-significant (*P* = 0.441) (Figure 3B). The sensitivity was 0.917, specificity 0.810, positive predictive value 0.524, negative predictive value 0.977, and overall accuracy 0.829. Decision curve analysis confirmed clinical utility (Figure 3C).

### 3.4 Nomogram and its clinical application

**FIGURE 4:**
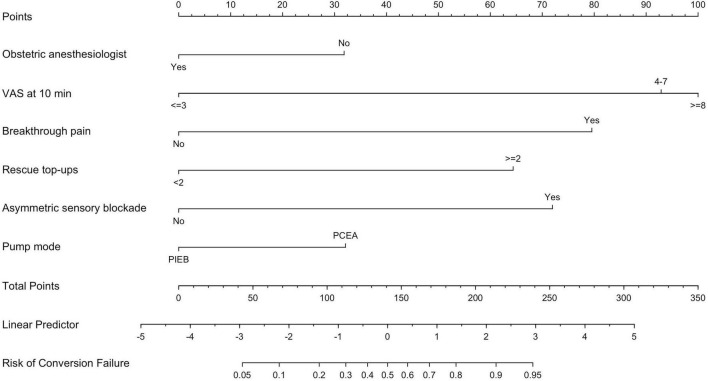
Nomogram for predicting failed conversion from epidural labor analgesia to cesarean epidural anesthesia. Variables include: obstetric anesthesiologist (yes vs. no), VAS 10 min post-labor analgesia (≤3, 4–7, ≥8), breakthrough pain (yes vs. no), rescue epidural top-ups (<2 vs. ≥2), asymmetric sensory blockade (yes vs. no), and pump mode (PIEB vs. PCEA). Instructions: For each patient, locate the value on each variable axis, draw a vertical line to the “Points” scale to assign points, sum all points to obtain “Total Points,” and draw a vertical line to the “Risk” scale to obtain the predicted probability of conversion failure.

A nomogram was constructed based on the final logistic regression model, incorporating the six retained predictors (Figure 4). The variables include: obstetric anesthesiologist (yes vs. no), VAS 10 min post-labor analgesia (≤3, 4–7, ≥8), breakthrough pain (yes vs. no), rescue epidural top-ups (<2 vs. ≥2), asymmetric sensory blockade (yes vs. no), and pump mode (PIEB vs. PCEA). Each variable was assigned a weighted score proportional to its effect size, with individual points summed to calculate a total risk score ranging from 0 to approximately 350 points, corresponding to predicted failure probabilities from 5% to 95%.

### 3.5 Clinical application of the nomogram

The nomogram is designed for use in non-immediate (Category 2) cesarean sections, where the 30–75 min window permits individualized anesthesia planning. It should be applied at the time of cesarean decision based on clinical data available during labor analgesia.

Step-by-step use:

1. Assign points for each risk factor based on the patient’s status
2. Sum the points to obtain total score
3. Read the corresponding predicted failure probability from the scale

Example: A patient managed by a non-obstetric anesthesiologist (approximately 32 points), with VAS at 10 min post-analgesia ≤ 3 (0 points), breakthrough pain present (approximately 80 points), ≥2 rescue top-ups (approximately 64 points), no asymmetric sensory blockade (0 points), and PIEB pump mode (0 points) would have a total nomogram score of approximately 176 points, corresponding to an estimated failure probability of approximately 74%. In this scenario, the anesthesiologist should proactively prepare spinal anesthesia equipment or consider early escalation according to maternal and fetal urgency.

Risk stratification and clinical actions:

Low risk (<20%): Proceed with standard epidural conversion.

Moderate risk (20%–50%): Prepare spinal anesthesia equipment at bedside.

High risk (>50%): Consider primary spinal anesthesia or early conversion to general anesthesia; inform senior anesthesiologist.

This approach aims to avoid the delay, maternal anxiety, and potential fetal compromise associated with failed conversion attempts in urgent situations.

## 4 Discussion

In this study, we developed and externally validated a prediction model for failed conversion from epidural labor analgesia to cesarean epidural anesthesia. From an initial set of 38 candidate variables identified through literature review, the nomogram incorporates six routinely available clinical variables: obstetric anesthesiologist involvement, VAS at 10 min post-analgesia, breakthrough pain, frequent rescue epidural top-ups, asymmetric sensory blockade, and pump mode (PIEB vs. PCEA). The model demonstrated good discrimination (AUROC 0.89 in both cohorts) and acceptable calibration (Hosmer-Lemeshow *P* = 0.890 and 0.441), with bootstrap validation suggesting limited optimism (optimism-corrected AUROC 0.88).

### 4.1 Risk factor analysis

The retained predictors should be interpreted as clinically overlapping indicators of epidural catheter performance rather than as fully independent biological mechanisms. High VAS at 10 min, breakthrough pain, repeated rescue top-ups, and asymmetric sensory blockade all reflect early manifestations of inadequate epidural function, such as catheter migration, unilateral spread, insufficient block density, or uneven local anesthetic distribution. This overlap likely contributes to the model’s strong discrimination. The clinical value of the nomogram is therefore not that it discovers unexpected causal pathways, but that it converts the anesthesiologist’s qualitative impression of poor catheter function into explicit, measurable bedside criteria. Obstetric anesthesiologist involvement remained protective (OR 0.38, 95% CI 0.19–0.77), and PIEB pump mode was also associated with lower odds of conversion failure (OR 0.38, 95% CI 0.19–0.75), consistent with the concept that specialized obstetric anesthesia practice and intermittent bolus delivery may improve labor epidural quality (18, 19).

A recently published nomogram by Zheng et al. also explored risk factors for conversion failure, though their model included different predictor variables, highlighting the need for population-specific validation (14). Additionally, emerging evidence suggests that chorioamnionitis may be associated with increased risk of conversion failure, possibly due to inflammatory changes affecting local anesthetic efficacy (20).

### 4.2 Clinical context and comparison with literature

In our institutional practice, Category 1 cesarean sections (immediate threat to life, delivery within 5 min) routinely receive general anesthesia and were excluded. The nomogram is intended for Category 2 cesarean sections (maternal or fetal compromise requiring delivery within 30–75 min), where the available time window permits individualized anesthesia planning. Although Category 2 allows more time than Category 1, the perceived urgency may still influence anesthesiologists’ decision-making and contribute to conversion failure.

Our conversion failure rate of 18.5% in the model-development cohort and 18.6% in the external validation cohort is consistent with the 0%–21% range reported in the literature (7, 8) and aligns with recent retrospective cohort studies in similar time-constrained clinical settings (12, 13, 15). The wide confidence intervals for some predictors (e.g., asymmetric blockade OR 8.71, 95% CI 2.33–32.52; VAS ≥ 8 OR 20.24, 95% CI 2.27–180.69) reflect the relatively low prevalence of these factors and warrant cautious interpretation.

The proposed nomogram offers several practical advantages. First, all variables are routinely collected during labor analgesia, enabling real-time bedside use. Second, external validation from a separate institution supports transportability within similar practice settings. Third, the tool provides a structured way to quantify early signs of poor epidural function and translate them into risk-stratified anesthesia planning. Fourth, systematic evaluation of 38 candidate variables helps clarify why a compact group of clinically available indicators can support decision-making.

## 5 Limitations

This study has several limitations. First, although external validation was performed, the model was developed from two institutions with similar practice patterns; validation in diverse populations is needed. Second, the retrospective design may introduce selection bias. Third, data on exact decision-to-delivery time were not available; future studies should examine whether time pressure modifies predictive performance. Fourth, abdominal circumference and biparietal diameter were classified based on gestational age-specific reference ranges, which may vary across different standards. Fifth, despite incorporating 38 potential predictors, unrecognized confounding factors may persist. Sixth, the relatively small sample size in certain subgroups resulted in wide confidence intervals, necessitating larger studies for confirmation. Seventh, our epidural extension regimen used 2% lidocaine without routine epinephrine or bicarbonate, and the initial 10 mL test/loading dose may be more conservative or slower in onset than protocols used elsewhere; differences in local anesthetic formulation, alkalinization, vasoconstrictor use, and dosing volume may partly influence the observed conversion failure rate. Finally, our institutional policy mandates routine general anesthesia for Category 1 cesarean sections, meaning these highly urgent cases were excluded from our cohort. While this ensures cohort homogeneity, it may limit the external validity and generalizability of our nomogram for institutions that routinely attempt rapid epidural extension even for Category 1 deliveries.

## 6 Conclusion

We developed and externally validated a clinical nomogram predicting failed conversion from epidural labor analgesia to cesarean anesthesia. Incorporating six routinely available variables, the model demonstrated good discrimination and acceptable calibration across two cohorts. This tool may help anesthesiologists quantify early signs of inadequate epidural function and support individualized planning at the time of cesarean decision.

## Data Availability

The original contributions presented in the study are included in the article/Supplementary material. Further inquiries can be directed to the corresponding author.

## Supplementary material

Supplementary Table 1 Baseline characteristics of all 38 candidate variables in the model-development and validation cohorts.
